# Smart-AMPs: Decorated Nanostructured Lipid Carriers for Improved Efficacy of Antimicrobial Peptides in Chronically Infected Burn Wounds

**DOI:** 10.3390/pharmaceutics17081039

**Published:** 2025-08-10

**Authors:** Daniela Müller, Laura Nallbati, Cornelia M. Keck

**Affiliations:** Institute for Pharmaceutics and Biopharmaceutics, Philipps-Universität Marburg, Robert-Koch-Str. 4, 35037 Marburg, Germany; daniela.mueller@pharmazie.uni-marburg.de (D.M.); laura.nallbati@pharmazie.uni-marburg.de (L.N.)

**Keywords:** burn wound infection, antimicrobial peptides (AMPs), nanostructured lipid carriers (NLCs), ex vivo porcine infected wound model, smart-AMPs

## Abstract

**Background/Objectives**: Burn wound infections present significant clinical challenges due to multidrug-resistant pathogens and the limitations of traditional antimicrobials. While antimicrobial peptides (AMPs) have broad-spectrum effectiveness, their instability in wound environments limits their use. This study compares properties of AMP-decorated nanostructured lipid carriers (NLCs) to free AMPs, focusing on their dermal penetration, retention, and antimicrobial efficacy in simulated ex vivo burn wound models. **Methods**: AMP-decorated NLCs (smart-AMPs) were produced by electrostatic and hydrophobic surface adsorption and characterized regarding their size, zeta potential, and physical short-term stability. The distribution of AMPs within the wounds was evaluated using an ex vivo porcine ear model with various wound types. The antimicrobial efficacy was assessed by monitoring the bioluminescence of *Aliivibrio fischeri* as a live bacterial marker for 24 h. **Results**: The size and zeta potential measurements confirmed the successful formation of smart-AMPs. The dermal penetration of AMPs was influenced by the type of wound and the type of AMP formulation (free AMPs vs. smart-AMPs). In the chronically infected burn wounds, which were characterized by the formation of a biofilm in a protein-rich wound fluid, the smart-AMPs resulted in a 1.5-fold higher and deeper penetration of the AMPs, and the antimicrobial activity was 6-fold higher compared to the free AMPs. **Conclusions**: smart-AMPs present an innovative approach for treating chronic, biofilm-associated wounds more efficiently than the current treatment options.

## 1. Introduction

Burn wounds represent a significant global health concern, accounting for substantial morbidity, mortality, and healthcare costs, particularly in low- and middle-income countries. Burn wounds cause about 180,000 deaths and 11 million injuries annually, mostly in low- and middle-income countries where child mortality rates are over seven times higher than in wealthier nations [1]. When burn injuries compromise the integrity of the skin, the body’s primary barrier to infection is lost, predisposing the wounds to microbial colonization and subsequent infection. Infected burn wounds can delay healing, increase the risk of sepsis, and complicate clinical outcomes, making their prompt and effective treatment a critical component of burn wound care [2].

The microbial landscape of burn wounds is dynamic and often polymicrobial, with the common pathogens including *Staphylococcus aureus* (including MRSA), *Pseudomonas aeruginosa*, *Acinetobacter baumannii*, and various fungal species. The progression from colonization to infection is influenced by factors such as the burn depth, wound size, immune status, and nosocomial exposure. As antimicrobial resistance becomes increasingly prevalent, especially in hospital-acquired strains, traditional treatment approaches face growing challenges [2,3].

The current strategies for managing infected burn wounds integrate systemic and topical antimicrobial therapies; surgical debridement; and advanced biomaterial-based wound dressings, like hydrogels, foams, or films designed to optimize the local wound environment [4,5].

Another strategy for the improved treatment of infected burn wounds is the use of antimicrobial peptides (AMPs) [6,7]. AMPs are naturally occurring or synthetic short peptides with broad-spectrum activity against bacteria, fungi, and some viruses. Their mechanisms—such as membrane disruption and immunomodulation—make them effective against multidrug-resistant organisms commonly found in burn infections, including *Pseudomonas aeruginosa* and *Staphylococcus aureus*. However, their limitations, such as enzymatic degradation and poor stability in vivo, have hindered their clinical applications [8,9].

Various formulation strategies, including coupling to polymers, peptide self-assembly, and the use of polymeric scaffolds and wound dressings, have already been used to overcome these challenges and to improve the efficacy of AMPs in infected wounds [10].

In this study, we decorated nanostructured lipid carriers (NLCs) with AMPs and investigated the effect of AMP-immobilization on the dermal penetration efficacy and antimicrobial efficacy of the AMPs in infected wounds.

**Figure 1:**
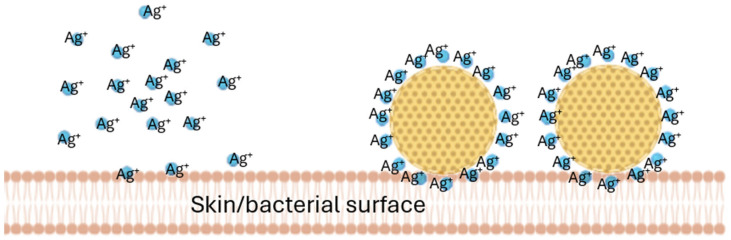
Principle of surface-decorated nanostructured lipid carriers. The surface decoration of nanostructured lipid carriers (NLCs) with cationic molecules, like positively charged silver ions, is achieved through the electrostatic, non-covalent adsorption of the silver molecules onto the negatively charged nanocarrier surfaces (modified from [14]).

NLCs are a well-known drug delivery system that can be used to improve the dermal penetration of active compounds [11,12,13]. After dermal application, NLCs form a long-lasting film on top of the skin (invisible patch) which protects the skin, hydrates the skin, and enables the improved dermal penetration of the active compounds of the NLCs. NLCs typically possess a negative surface charge (zeta potential) to which positively charged molecules can adhere. The adherence of positively charged molecules onto the surfaces of the NLCs immobilizes them and creates locally higher concentrations of the compound when compared to the freely dissolved molecules. This principle has already been exploited for silver ions by Schwabe et al [13] (Figure 1).

This locally high concentration gradient of silver ions, bound to NLCs, was then shown to improve the antimicrobial activity of the silver [14,15,16]. AMPs, which bear a positive charge similar to that of silver, were therefore considered to have a similar capacity to bind to negatively charged NLC surfaces. The objective of this study was, therefore, to develop an AMP-decorated NLC complex (smart-AMP) to assess whether the principle of the silver–nano-lipid complex could also be exploited with AMPs. Hence, the study aimed to compare the penetration efficacy and resulting antimicrobial activity of free AMPs to AMP-decorated NLCs in different types of wounds.

## 2. Materials and Methods

### 2.1. Materials

The positively charged AMP consisted of 21 amino acids (Iris Biotech GmbH, Marktredwitz, Germany) with the sequence FALGAVTKVLPKLFCLITRKC [17], to which an N-terminal fluorescent label (Fluorescein isothiocyanate (FITC), Sigma-Aldrich, Taufkirchen, Germany) was added.

Nanostructured lipid carrier (NLC) consisted of 8% _(w/w)_ cetyl palmitate (cetyl palmitate 15, CP, Caesar & Loretz GmbH, Hilden, Germany), 2% _(w/w)_ medium-chain triglycerides (Miglyol^®^ 812, Caesar & Loretz GmbH, Hilden, Germany), 1% _(w/w)_ C8-C16 alkylpolyglucoside (Plantacare^®^ 818, BASF AG, Germany), and 89% _(w/w)_ purified water (freshly obtained using a PURELAB Flex 2 water purification system (ELGA LabWater, Veolia Water Technologies GmbH, Germany)).

The bioluminescent bacteria *Aliivibrio fischeri (A. fischeri)* strain NRRL-B-11177 was obtained in freeze-dried form from the Leibniz Institute DSMZ-German Collection of Microorganisms and Cell Cultures GmbH (Braunschweig, Germany).

### 2.2. Methods

#### 2.2.1. Synthesis and Antimicrobial Activity of Fluorescence-Labeled AMP

The AMP was synthesized at a 0.1 mM scale using a microwave-assisted peptide synthesizer LibertyBlue™ (CEM GmbH, Kamp-Lintfort, Germany) by 9-Fluorenylmethoxycarbonyl (fmoc) chemistry [18]. For successive couplings, the protected amino acids dissolved in N’N’-dimethylformamide (DMF) were added at a 5-fold excess. Fmoc deprotection was affected with 20% piperidine in DMF. The peptide was cleaved from the resin using a mixture of trifluoroacetic acid (TFA), thioanisole, water, and 3,6-dioxa-1,8-octanedihiol (DODT) (92.5%, 2.5%, 2.5%, 2.5% _(v/v)_), precipitated in tert-butyl methyl ether (−80 °C). The purity of the AMP was proven by a reversed-phase HPLC on an analytical column (Phenomenex^®^; Aeris ™ 2.6 µm Peptide XB-C18 100 Å LC column 150 × 2.1 mm) using a 10–40% CH_3_CN gradient with 0.05% TFA in 15 min at a 1 mL/min flow rate. The peptide showed a purity > 95% [17].

To enable the tracking of the AMP within the wound tissue, the AMP was fluorescently labeled with fluorescein isothiocyanate (FITC) (Sigma-Aldrich, Taufkirchen, Germany) [19]. For this, the AMP was dissolved in 0.1 M sodium carbonate buffer (pH 9.0) at a concentration of 1–5 mg/mL. FITC was dissolved in dimethyl sulfoxide (DMSO; Sigma-Aldrich, Taufkirchen, Germany) and added dropwise to the peptide solution at a 5:1 molar ratio (FITC–peptide). The reaction was incubated for 2 h at room temperature in the dark under gentle agitation. The unreacted FITC was removed by using ultrafiltration units (Amicon^®^ Ultra-4 ultrafiltration, 3 kDa MW Merck-Millipore, Darmstadt) that were centrifuged at 4300× *g* for 30 min. The retentate was then resuspended in 1 mL water and stored at 4 °C ± 2 °C in the dark until further use. The antimicrobial activity of the FITC-labeled AMP was confirmed according to the process described in Section 2.2.8 (see below).

#### 2.2.2. Production of NLC

The lipids (cetylpalmitate and medium-chain triglycerides) were combined, melted, and heated to 75 °C in a beaker while stirring. Meanwhile, the aqueous phase (water and surfactant) was prepared by adding the surfactant to the water and heating the aqueous phase to 75 C. Subsequently, the hot water phase was added to the hot lipid phase while agitating, and the resulting hot pre-emulsion was high-speed stirred at 24,000 rpm for 30 s (Ultra Turrax T25, IKA Werke GmbH & Co. KG, Staufen, Germany). Subsequently, high-pressure homogenization was performed at 75 °C and 500 bar for three cycles (LAB 40, APV Gaulin GmbH, Wentorf bei Hamburg, Germany). The obtained formulation was then cooled in an ice bath. After cooling, it was poured into Falcon tubes and stored at room temperature until further use [20].

#### 2.2.3. Production of Smart-AMP: Decoration of NLC with AMP

The surface decoration of the NLC with the AMP was conducted at a ratio of 0.1% _(w/w)_ NLC to 0.16% _(w/w)_ peptide. For this, 5 mg of NLC and 8 mg of peptide were weighed into a centrifuge tube and made up with 5 mL of purified water. The suspension was incubated at 300 rpm for 3 h at room temperature on a shaker. Subsequently, the suspension was transferred to ultrafiltration units (Amicon^®^ Ultra-4 ultrafiltration, 30 kDa MW Merck-Millipore, Darmstadt, Germany) and centrifuged (4300× *g*) for 20 min. The retentate was resuspended in 1 mL ultrapure water and diluted 1:1.6 to a concentration of 1 mg/mL with purified water. The product was an AMP-decorated NLC that was kept at 4 °C ± 2 °C until further use.

#### 2.2.4. Physico-Chemical Characterization of NLC and AMP-Decorated NLC

The physico-chemical characterization of the non-decorated NLC and the AMP-decorated NLC was performed by measuring the particle size and the zeta potential (ZP) by using dynamic light scattering (DLS) and laser Doppler anemometry (LDA), respectively. In addition, light microscopy was performed to detect possible larger particles, i.e., agglomerates, that would not be visible by DLS, the upper detection limit of which is approx. 3 µm [20].

DLS analyzes the hydrodynamic diameter of a formulation (z-average) and the polydispersity index (PdI) as a parameter for the width of the size distribution. The PdI can range from 0 to 1, where formulations with PdI values < 0.2 are assumed to present a sufficiently narrow size distribution, whereas larger values represent a broad size distribution, which is associated with lower physical stability [20]. The DLS measurements were performed using a Zetasizer NanoZS (Malvern-Panalytical, Kassel, Germany). The results were obtained as the average of 10 acquisitions analyzed using the general-purpose mode of the instrument’s on-board analytical software.

The ZP analysis was performed using a Zetasizer NanoZS (Malvern-Panalytical, Germany) in its M3-PALS setup. Laser Doppler anemometry (LDA) determines the electrophoretic mobility that is subsequently converted into the zeta potential using the Helmholtz–Smoluchowski equation [21]. The particles were analyzed in purified water adjusted to a conductivity of 50 μS/cm at 20 °C (conductivity water, CW). Additionally, to assess how tightly the AMP was bound to the surface of the NLC, the particles were also analyzed in their original dispersion medium (OM). For this, the formulation was dispersed in 1% _(w/w)_ surfactant solution prior to each measurement. The difference in the ZP between the OM and water was used as a measure for the binding efficacy (BA), where small differences indicate the tight binding of the AMP (none or only small proportions of the AMP are washed off when the formulation is dispersed in pure water), whereas larger differences indicate that the AMP is easily washed off from the NLC upon dilution with water [22,23].

The LM images were taken using an Olympus BX53 light microscope (Olympus Corporation, Tokyo, Japan) equipped with an Olympus SC50 CMOS color camera (Olympus Soft Imaging Solutions GmbH, Münster, Germany). Magnifications ranging from 40-fold to 1000-fold were used to observe the samples. Small magnifications were used to oversee the samples and to observe possible agglomerates quickly. Larger magnifications were used to access more detailed information about the particles.

#### 2.2.5. Preparation of *A. fischeri* Culture

*A. fischeri* was cultured overnight using growth medium 1856 according to the guidelines of the Leibniz Institute DSMZ-German Collection of Microorganisms and Cell Cultures GmbH (Braunschweig, Germany). The composition of the growth medium used is given in Table 1. After inoculation, the bacteria were allowed to incubate overnight at room temperature on a shaker at 150 rpm (KS 15 A, Edmund Bühler GmbH, Hechingen, Germany).

#### 2.2.6. Creation and Characterization of Ex Vivo Burn Wound Types

Wounds were created on fresh pig ears. The characterization of the wounds and the determination of the AMP penetration into the wounds was performed following the protocol used for testing the ex vivo dermal penetration efficacy of active compounds in porcine skin with a subsequent digital image analysis [24,25]. For the experiments, fresh pig ears were obtained from a local slaughterhouse and used within 4 h after slaughter [24,25]. In the first step, the ears were washed and carefully dabbed dry by using paper towels. The transepidermal water loss (TEWL) values were then measured by using a Tewameter^®^ TM 300 (Courage & Khazaka electronic GmbH, Cologne, Germany) to verify skin integrity. Only ears with a TEWL ≤ 10 were included in the study. In the second step, standardized burn wounds were created on the ears with an electric soldering iron (60 W, Ockered, China) with a round copper tip (Ø 5 mm). The burning temperature was 350 °C, the burning time was 30 s, and no pressure was applied to the skin during the burning process.

**Figure 2:**
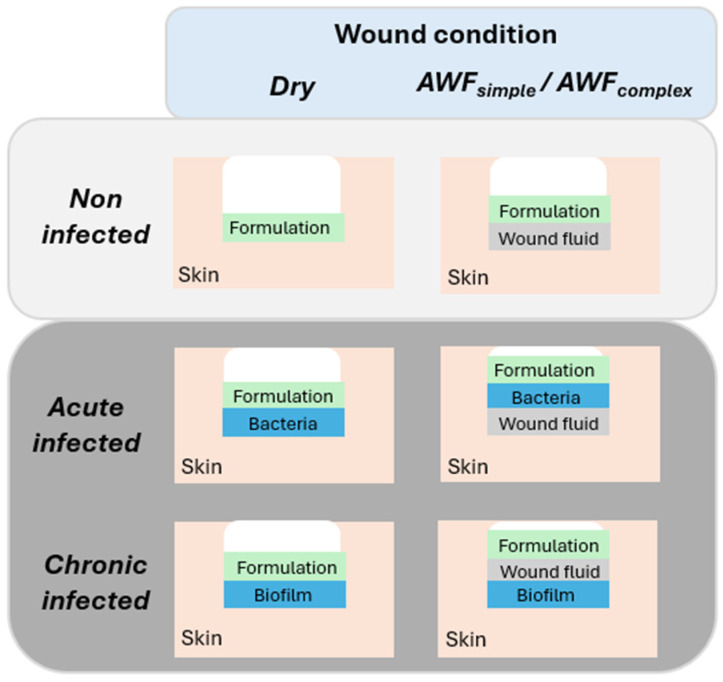
Schematic representation of different types of wounds and wound conditions used in this study.

To simulate different types of wounds, different procedures for infecting the wounds with bacteria and different types of wound fluids were used (Table 1). To mimic dry and wet wounds, the wounds were either left dry or were filled with 2 µL simple artificial wound fluids (AWFs) (Figure 2, Table 2). Two types of AWFs were used (AWF_simple_ and AWF_complex_). The AWF_simple_ consisted of 0.68 g of NaCl, 0.35 g of NaH_2_PO_4_, 2.5 g of NaHCO_3_, and 0.22 g of KCl in 100 mL and had a pH of 8.0 ± 0.2 [26]. The AWF_complex_ was similar to the AWF_simple_, but contained 3.4% bovine serum albumin (BSA, protease free, lyophilized fraction V, purity ≥ 98.5%, Sigma-Aldrich, Taufkirchen, Germany) that was added to mimic the wound fluid proteins and to allow for the investigation of protein binding and interaction with the AMP [26].

The wounds’ infection with the bacteria was performed by adding 2 µL of an overnight grownup *A. fischeri* culture (c.f. 2.2.5) to the wounds. The different types of wounds (acute infected and chronic infected) were created by adding the bacteria either prior to or after the filling of the wounds with the AWF (Figure 2, Table 2). The acute infected wounds mimicked conditions where bacteria are homogenously distributed within a wound and wound fluid. They were obtained by adding the bacteria culture after the addition of the AWF to the wounds. The chronically infected wounds mimicked wounds where bacteria already form a biofilm on the bottom of a wound. To allow for the formation of such a biofilm, the chronic wounds were obtained by adding the *A. fischeri* culture to freshly burned wounds. The AWF was then added after a 60 min resting at room temperature. For standardization, after their final formation, all the wounds were rested for 30 min at room temperature until further use.

#### 2.2.7. Determination of AMP Penetration Efficacy into Different Types of Wounds

A 2 µL AMP or AMP-decorated NLC sample (0.16% _(w/w)_) in purified water was applied onto the ex vivo wounds and allowed to penetrate for 2 h at room temperature. Afterwards, punch biopsies were taken (Ø 13 mm) from each sample. The biopsies were embedded in TissueTek^®^ (Sakura Finetek Europe B.V., Alphen aan den Rijn, The Netherlands), immediately frozen, and stored at −20 °C until further use. The experiments were performed in triplicate.

In the next step, the punch biopsies were cut into 20 µm thick vertical skin sections using a cryomicrotome (Frigocut 2700, Reichert-Junk, Nußloch, Germany). The skin cuts were placed onto microscopic slides and examined by using inverted epifluorescence microscopy (Olympus CKX53, Olympus Deutschland GmbH, Hamburg, Germany). The images were taken with an Olympus DP22 color camera (Olympus, Hamburg, Germany) attached to the fluorescence microscope (FM). All the images were taken at 40-fold magnification using a DAPI HC filter block system (460–490 nm (BP); dichroic mirror: 500 nm; emission filter: starting at 500 nm (LP)). The intensity of the fluorescent light was kept at 50% and the exposure time was 50 ms. All the settings were kept constant throughout the entire study [25].

The subsequent digital image analysis of the images obtained was used to determine the amount of penetrated AMP semiquantitatively. For this, the images were subjected to an automated RGB threshold using ImageJ v1.8.0, which subtracted the autofluorescence of the skin from the images [24,25]. From these thresholded images, the mean gray value per pixel (MGV/px) was obtained, which served as a semiquantitative measure of the total amount of permeated (TAP) AMP. In addition, the mean permeation depth (MPD) was measured using the software’s scaling function. The mean wound depth (MWD) was measured similarly to the MPD [24,25].

#### 2.2.8. Determination of Antimicrobial Activity in Infected Ex Vivo Burn Wounds

The antibacterial efficacy was evaluated by monitoring the bioluminescence of the *A. fischeri* in the wounds over time. For this, punch biopsies (Ø 13 mm) of the infected wounds (c.f. 2.2.6) were collected and placed in 24-well plates. The wounds were then treated with 2 µL of the respective AMP formulation. The non-infected wounds and non-AMP-treated wounds served as the controls. The antimicrobial activity was measured by monitoring the bioluminescence of *A. fischeri* over time using a TECAN Infinite 200 PRO microplate reader (Tecan Group, Männedorf, Switzerland) in top-read mode. The measurements were taken immediately after the treatment and after 1 h, 3 h, 6 h, 15 h, 20 h and 24 h. In between the measurements, the samples were kept at room temperature and protected from light with aluminum foil. All the experiments were performed in triplicate.

#### 2.2.9. Statistical Analysis

All the results were expressed as the mean ± standard deviation (SD), unless otherwise described. The descriptive statistics were calculated using JASP software (Version 0.19.3, University of Amsterdam, Amsterdam, The Netherlands) [27]. The statistical differences between the mean values were evaluated with JASP software (Version 0.19.3) and by using GraphPad Prism 10 software (Version 10.5.0, GraphPad Software, Boston, USA). The results were tested for normal distribution using the Shapiro–Wilk test, while the variance homogeneity was analyzed using Levene’s test. For the normally distributed data, the means were compared by a one-way ANOVA, and in the case of variance heterogenicity, a Welch correction was applied. Furthermore Games–Howell, Tukey’s, and Dunn post hoc tests were performed for the comparison of the mean values with each other. *p*-values < 0.05 were considered statistically significant. Significant differences among the different mean values were indicated by * *p* < 0.05, ** *p* < 0.01, and *** *p* < 0.001.

## 3. Results and Discussion

### 3.1. Synthesis and Antimicrobial Activity of Fluorescence-Labeled AMP

**Figure 3:**
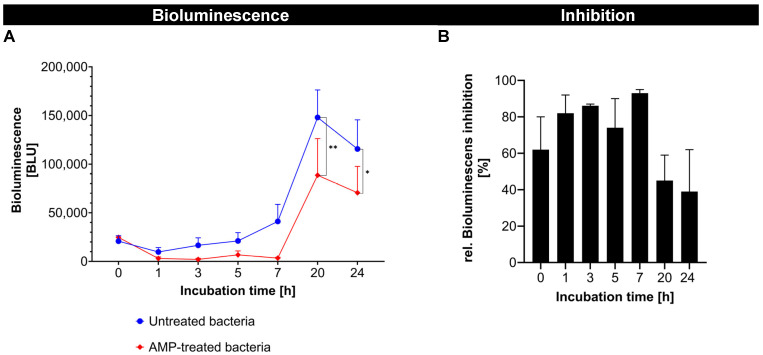
Effect of AMP treatment on bioluminescence of *A. fischeri* over time. (**A**) Absolute bioluminescence levels (in BLU) of untreated and AMP-treated bacteria. (**B**) Relative bioluminescence inhibition (%) in AMP-treated bacteria normalized to the untreated bacteria over time (* *p* < 0.05; ** *p* < 0.01).

In the first part of the study, fluorescence-labeled AMP was successfully synthesized and showed a strong antibacterial effect against *A. fischeri*, which was shown by a significant reduction in the bioluminescence of *A. fischeri* over time (Figure 3A). The antimicrobial activity of the fluorescence-labeled AMP showed a fast onset and reached its maximum (>90%) after about 6 h. After 15 h, a noticeable decline was observed, indicating that the antimicrobial effect was not persistent (Figure 3B). The reduction in antimicrobial activity after 15 h was likely due to the degradation or inactivation of the free AMP in the bacterial environment. AMPs in general are known to have a limited stability, being susceptible to proteolytic degradation by bacterial enzymes and/or to physicochemical changes, such as structural rearrangements or aggregation in complex biological media [28]. Despite this, the results confirm that the FITC-labeled AMP retained an antimicrobial activity comparable to its native, unlabeled form [17]. This validates its use as a functional model of AMP for further studies.

### 3.2. Physico-Chemical Characterization of Non-Decorated NLC and AMP-Decorated NLC

**Figure 4:**
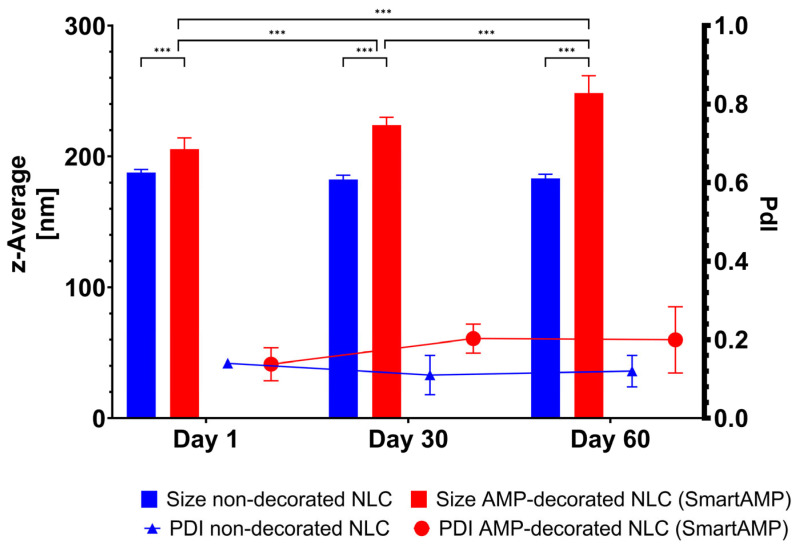
Particle size and PdI of non-decorated and AMP-decorated NLC (smart-AMP) analyzed by DLS (*** *p* < 0.001).

**Figure 5:**
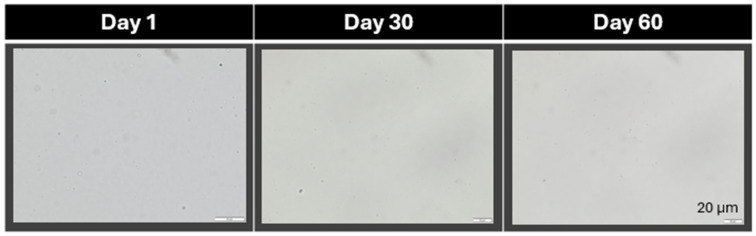
Light microscopic images of AMP-decorated NLC during stability testing monitored over 60 days. The images prove the absence of larger micrometer particles that could have occurred if the NLC had agglomerated. Hence, the NLC remained physically stable during the time of storage (600-fold magnification).

The non-decorated NLC possessed a size below 200 nm and a PdI below 0.2. The size remained unchanged during short-term storage (Figure 4). These results are in line with previous data [20]. The AMP decoration of the NLC led to an increase in size to about 210 nm, indicating that the AMP was successfully adsorbed onto the surface of the NLC. The size increased during storage. However, light microscopy did not reveal any signs of instability in or agglomeration of the particles (Figure 5). Therefore, the increase in size over time might not be due to instability, but due to the further adsorption of AMP onto the surface of the NLC.

**Figure 6:**
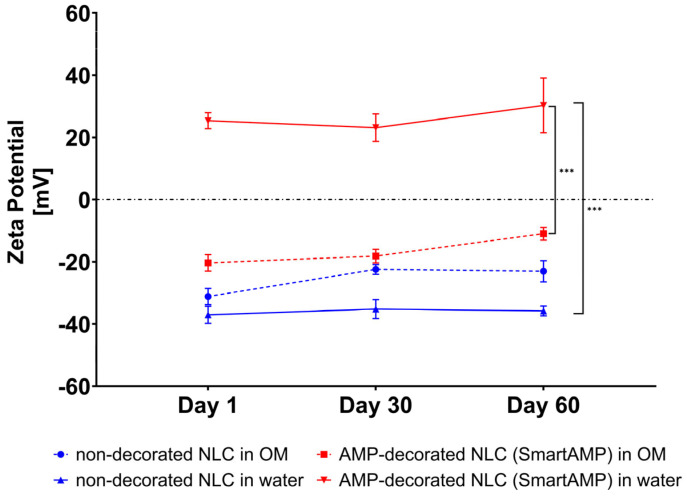
Zeta potentials of non-decorated and AMP-decorated NLC (smart-AMP) analyzed in original dispersion medium (OM, 1% Plantacare^®^ 818) and water (conductivity-adjusted water, 50 μS/cm) (*** *p* < 0.001).

A zeta potential analysis was used to provide further details on the adsorption of the AMP onto the surface of the NLC. The non-decorated NLC exhibited a strongly negative zeta potential of approximately −40 mV in water (Figure 6). After the addition of the AMP, the zeta potential increased to +25 mV, thus providing evidence that the binding of the cationic AMP onto the surface of the NLC was successful. Over time, the zeta potential of the AMP-decorated NLC increased, thus, like the size measurements, also indicating that the absorption of the AMP onto the surface of the NLC continued during storage. The measurements of the ZP in the original dispersion medium (OM) show that the measured ZP values were generally less negative or less positive when compared to the measurements in water. These effects were expected, as the water diluted the samples and washed off the surfactant and/or AMP that were only loosely bound to the surface of the NLC [29]. The relatively large differences between the ZP of the AMP-decorated NLC in water and the OM, indicate, however, that the AMP binding to the NLC was weak and that the AMP was easily washed off from the surface of the NLC when the formulation was diluted.

However, the measurements in the OM also show significant differences between the ZP of the non-decorated and AMP-decorated NLC, indicating that at least some parts of the AMP were tightly bound to the surface of the NLC, indicating that the smart-AMP was also maintained upon dilution with water and/or other media.

### 3.3. Creation and Characterization of Ex Vivo Burn Wound Types

**Figure 7:**
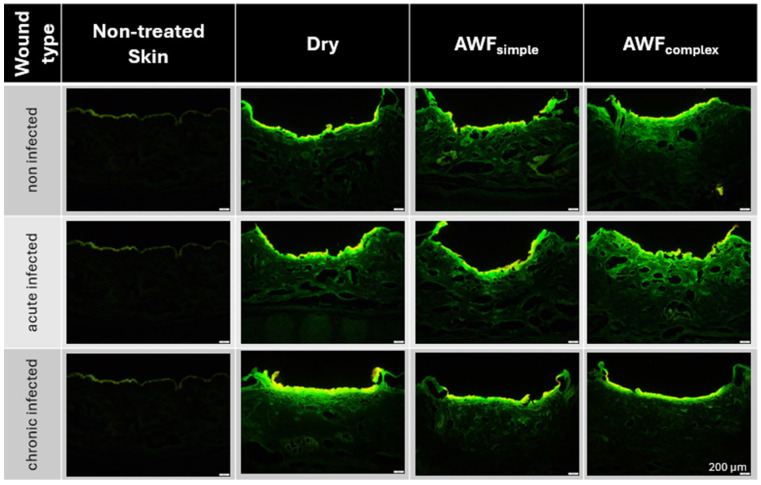
Fluorescence microscopic images of the different ex vivo porcine burn wounds (scale bar represents 200 µm).

**Figure 8:**
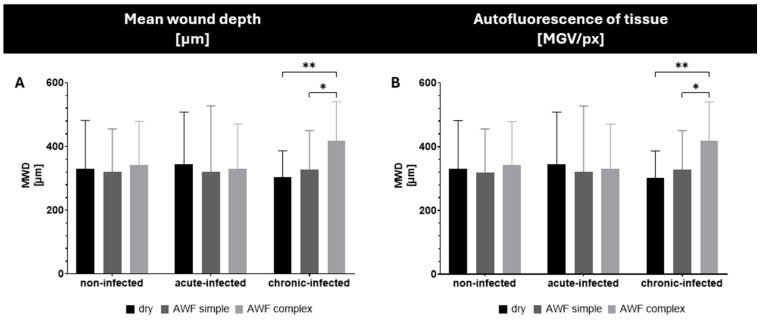
Influence of type of wound (type of infection and type of wound fluid) on (**A**) mean wound depth (MWD) and (**B**) autofluorescence of tissue (* *p* < 0.05; ** *p* < 0.01).

For the efficacy testing of the AMPs and smart-AMPs, different types of burn wounds were created (Figure 7). The burn wounds had a mean wound depth (MWD) of about 350 µm and the addition of artificial wound fluid did not alter this (Figure 8A). Only the chronically infected wounds with complex artificial wound fluid showed a slightly deeper MWD, which indicates a slightly deeper tissue destruction, likely driven by the metabolic activity of the biofilm-embedded bacteria that was facilitated by the protein-rich environment.

The burning of the skin not only created holes in the skin but also increased the autofluorescence of the burned skin areas (Figure 8B). Such an increase was expected and was due to the loss of water and the resulting increase in tissue density. The addition of the bioluminescent bacteria to the wounds also increased the autofluorescence of the wounds. The effect was most pronounced for the chronic wounds where the bacteria could form a dense biofilm on top of the wounds (Figure 7 and Figure 8).

The addition of AWF had no significant influence on the autofluorescence of the non-infected wounds. The results indicate the morphological stability of the non-infected wounds and their suitability as a reference model [26]. In the bacteria-infected wounds, the addition of AWF caused a slight increase in the autofluorescence of the acute infected wounds and a slight decrease in the chronically infected wounds (Figure 8B). The observed changes in the autofluorescence of the infected wounds upon addition of AWF indicate structural changes in the wound tissue and/or differences in the distribution of the bacteria within the wounds [30]. The addition of AWF to the dry, acute infected wounds seems to have increased the penetration depth of the bacteria. This effect is reasonable, because the AWF contains water that can hydrate and swell the skin, thus allowing for the easier penetration of the bacteria into deeper layers of the skin. In the chronic infected wounds, the effect was the opposite. Hence, the addition of AWF decreased the autofluorescence of the tissue and the bacteria penetration was rather less deep. Also, this effect is reasonable, because AWF can be considered to foster the growth of bacteria. This means that the biofilm of the chronically infected wounds became more localized and denser upon the addition of the AWF, which then resulted in a lesser penetration depth and less all-over autofluorescence of the skin biopsies. As each type of wound created represented a different type of real-world situation, all the kinds of wounds created in this part of the study were used for the subsequent studies, where the influence of the AMP’s immobilization onto the surface of the NLC on the penetration efficacy of the AMP and its antimicrobial activity was investigated.

### 3.4. Determination of AMP Penetration Efficacy into Different Types of Wounds

**Figure 9:**
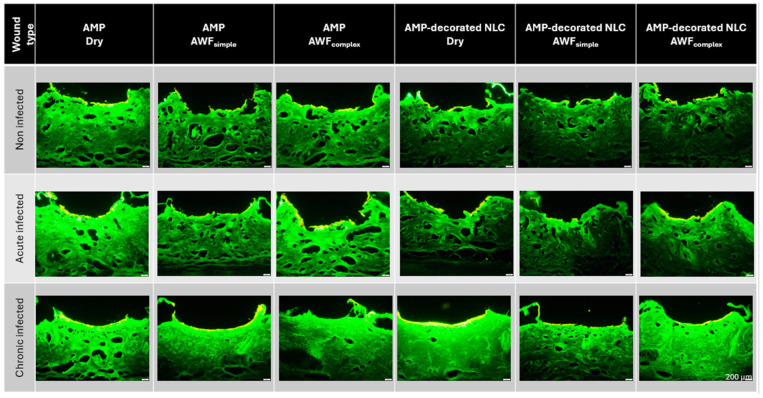
Fluorescence images of free AMP and AMP-decorated NLC (smart-AMP) after application to different types of wounds compared to untreated wounds (scale bars represent 200 µm).

**Figure 10:**
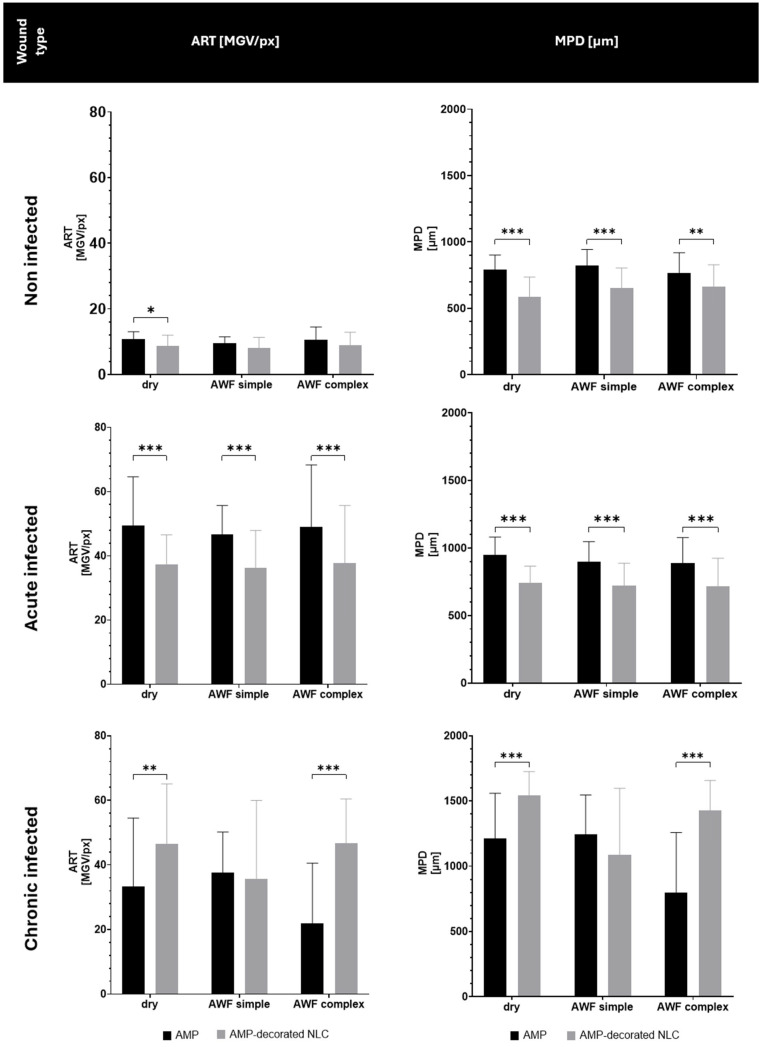
Comparison of dermal penetration of AMP and AMP-decorated NLC into ex vivo burn wound model for different infection states and wound conditions (* *p* < 0.05; ** *p* < 0.01; *** *p* < 0.001).

Both the type of wound and the binding of the AMP to the NLC influenced the penetration efficacy of the AMP (Figure 9 and Figure 10). In the non-infected wounds, and in the acute infected wounds, the binding of the AMP to the NLC resulted in a significant decrease in the penetration efficacy, which was about 15% for the non-infected wounds and about 22% for the acute infected wounds. In the chronic infected wounds, the binding of the AMP to the NLC increased the penetration efficacy by about 30% in the dry wounds and by about 50% in wounds that contained AWF (Figure 10).

The decreased penetration of the AMP bound to the NLC in the non-infected and acute infected wounds can be explained by the binding of the AMP to the NLC. The binding of the AMP reduced the number of free AMP molecules and thus, according to Fick’s law, the passive diffusion of the AMP into the skin was also reduced [31,32]. The addition of AWF had no or only very limited effects on the penetration efficacy. This outcome might be explained by the occurrence of different effects at the same time that were superimposed and/or might have cancelled each other out. The water in the AWF could be considered to have caused a slight increase in the penetration due to the hydration of the tissue. However, the negatively charged electrolytes and the protein within the AWF might have caused binding and/or agglomeration of the AMP, which could have hindered its penetration into the tissue.

The increase in the penetration of the AMP from the AMP-decorated NLC into the chronic wounds was expected and was caused by the locally higher concentration gradient of the AMP that was bound to the surface of the NLC (c.f. Figure 1). After its application to the dry wounds, the AMP-decorated NLC formed an invisible patch on top of the biofilm and interacted closely with the bacteria, which caused them to die. The death of the bacteria caused a disruption in the biofilm and provided an easy path for the AMP to penetrate into the skin. The effect was not visible in the wounds with simple AWF that only contained electrolytes, but was visible in the wounds that contained the complex AWF. Interestingly, the free AMP caused opposite penetration efficacies in the chronically infected wounds. Hence, the best penetration of the free AMP was found in the wounds with the simple AWF and the least penetration was found in the wounds with the complex AWF (Figure 10).

These effects can also be considered to be the result of the complex interplay between the AMP, wound tissue, AWF, and bacteria, and more research is needed to understand the effects observed in detail. However, the data already show that the binding of an AMP to an NLC can significantly change or even reverse the bio-efficacy of AMP in different types of wounds.

### 3.5. Determination of Antimicrobial Activity in Infected Ex Vivo Burn Wounds

**Figure 11:**
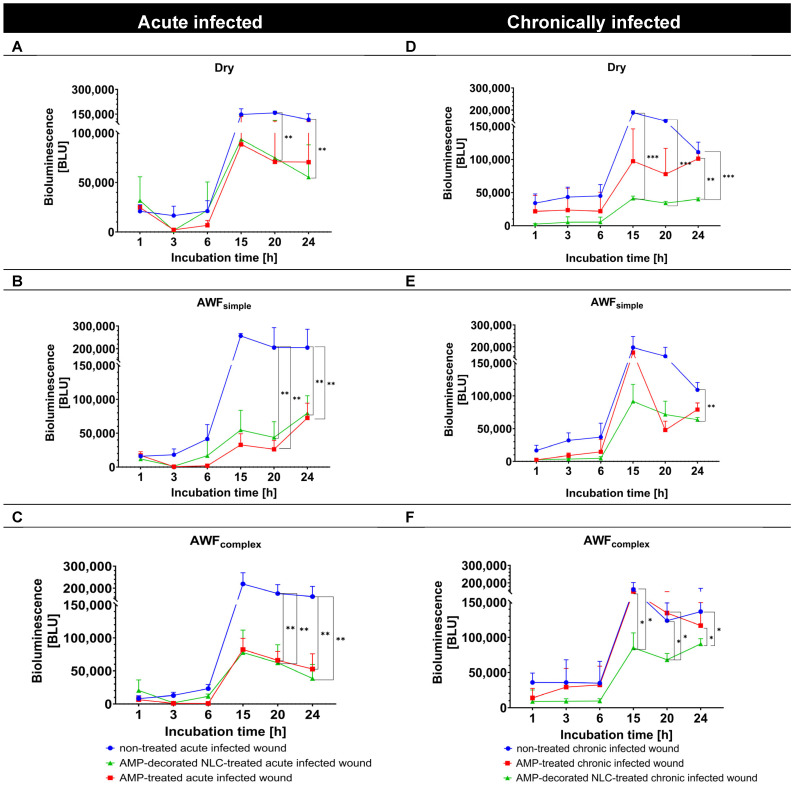
Effects of AMP and AMP-decorated NLC on bioluminescence of *A. fischeri* in acute infected (**A**–**C**) and chronically infected wounds (**D**–**F**) and different wound environments (* *p* < 0.05; ** *p* < 0.01; *** *p* < 0.001).

The antimicrobial efficacy of the AMP formulations in the wound models was evaluated in the next step by measuring the bioluminescence (BL) of *A. fischeri* over 24 h and comparing it to that for the untreated control wounds (Figure 11). In all the acute infected wound types, the untreated controls showed a consistent increase in *A. fischeri* bioluminescence over time, suggesting active bacterial growth. The bacterial growth curve of *A. fischeri* was characterized by an initial lag phase (0–6 h), followed by a log phase (6–15 h), and then continued in a stationary phase (15–24 h) (Figure 11, left).

In the chronically infected wounds, the course was similar, but the stationary phase seemed to be shorter, as the BL had already started to decline after 20 h (Figure 11, right), indicating the initiation of the dead phase after about 20 h. Based on these bacterial growth curves, the antimicrobial activity of the free AMP and AMP bound to the NLC could be judged.

In the dry, acute infected wounds, both the free AMP and AMP bound to the NLC efficiently reduced the BL of *A. fischeri* in the log phase. In the lag phase, the AMP-decorated NLC were more efficient at preventing the growth of the bacteria (Figure 11A). In the acute infected wounds with AWF, both the free AMP and AMP bound to the NLC were similarly efficient at reducing the BL of *A. fischeri* (about –50%) in both the log and the lag phase, respectively (Figure 11B,C).

**Figure 12:**
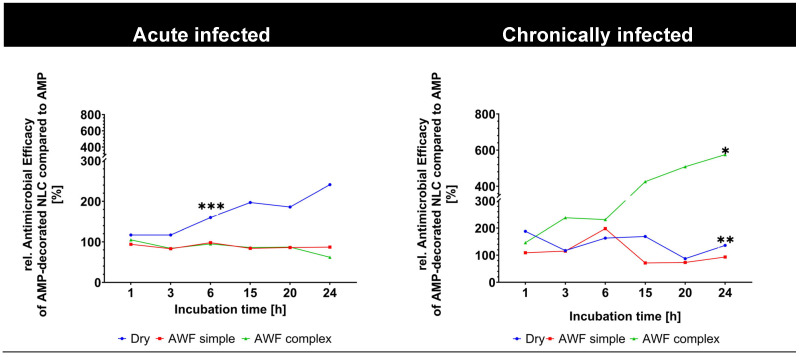
Determination of antimicrobial activity of AMP and AMP-decorated NLC in different types of wounds (* *p* < 0.05; ** *p* < 0.01; *** *p* < 0.001).

In the chronically infected wounds, the AMP-decorated NLC were more efficient at BL reduction than the free AMP (Figure 11D–F). The improved antimicrobial activity of the AMP bound to the NLC when compared to the free AMP was visible in all the different phases of the bacterial growth curve (log, lag, stationary, and death phases) and also for the different types of wounds (dry, simple, and complex AWF, Figure 11D–F). The improved antimicrobial effect of the AMP-decorated NLC was most pronounced in the wounds with the complex AWF and in the stationery and death phases, where it yielded an about 4–6-fold higher antimicrobial activity than the free AMP (Figure 11F and Figure 12).

The observed antimicrobial effects of the AMP in its different formulations are considered to be the result of a complex interplay between the AMP, bacteria, AWF, and wound tissue compounds, and more research is now needed to understand the effects in detail. AMPs and smart-AMPs can be seen as intermediate products and thus need further testing and their formulation into a final wound dressing, which could also have an important influence on the bio-efficacy of AMPs. Therefore, future studies should also focus on the development of functional wound dressings in which smart-AMPs are incorporated. In this way, highly effective and multifunctional treatments for chronically infected wounds could be realized. However, despite the complexity of the effects, the data clearly show the superior efficacy of AMPs bound to NLCs compared to free AMPs, which indicates that the use of smart-AMPs has great potential to treat chronically infected wounds more efficiently in the future.

## 4. Conclusions

A fluorescently labeled AMP with good antimicrobial efficacy against *A. fischeri* was synthesized and used as a model AMP in this study. The addition of a negatively charged NLC to the positively charged model AMP led to the formation of a smart-AMP, meaning an AMP-decorated NLC. The size analysis and zeta potential measurements confirmed the successful formation of a smart-AMP, and the penetration experiments as well as the antimicrobial efficacy tests demonstrated that the smart-AMP was more effective than the free AMP. Based on these findings, it is concluded that smart-AMPs are a promising formulation principle, particularly for treating chronically infected wounds.

## Figures and Tables

**Table 1:** Composition of cultivation medium 1856.

Ingredient	Amount
NaCl	30.00 g
NaH_2_PO_4_ × H_2_O	6.10 g
K_2_HPO_4_ × 3 H_2_O	2.75 g
MgSO_4_ × 7 H_2_O	0.204 g
(NH_4_)_2_HPO_4_	0.500 g
glycerin	3.00 mL
casein peptone	5.00 g
yeast extract	0.500 g
purified water	add 1000 mL
pH	adjusted to 7.0

**Table 2:** Different types of wounds used within this study.

Wound Type	Condition	Composition/Experimental Description	Infection Agent
Non infected wounds	Dry	No fluid added; wound tissue exposed to ambient air; represents a dehydrated, protein-free environment.	None
	AWF_simple_	Artificial wound fluid with inorganic salts only (NaCl, NaH_2_PO_4_, NaHCO_3_, KCl; pH 8.0); no proteins.	None
	AWF_complex_	AWF_simple_ + 3.4% bovine serum albumin; mimics protein-rich exudate.	None
Acute infected wounds	Dry	Infected wound without added fluid; early-stage infection under dry, non-exudative conditions.	*Aliivibrio fischeri* (bioluminescent, freshly diluted)
	AWF_simple_	Salt-based AWF added to infected wound; non-protein fluidic environment.	*Aliivibrio fischeri* (bioluminescent, freshly diluted)
	AWF_complex_	AWF_simple_ + albumin/fibrinogen; simulates acute infection with protein-rich exudate.	*Aliivibrio fischeri* (bioluminescent, freshly diluted)
Chronically infected wounds	Dry	Biofilm-based chronic infection without added fluid; impaired hydration and dense tissue matrix.	*Aliivibrio fischeri* (bioluminescent, pre-incubated)
	AWF_simple_	Inorganic salt solution in biofilm-infected tissue; simulates chronic wounds with minimal exudate.	*Aliivibrio fischeri* (bioluminescent, pre-incubated)
	AWF_complex_	Protein-enriched AWF in biofilm-infected tissue; models chronic, exudative, and biofilm-rich wound environments.	*Aliivibrio fischeri* (bioluminescent, pre-incubated)

## Data Availability

The data will be made available upon request.
